# Adsorption and Orientation of Human Islet Amyloid Polypeptide (hIAPP) Monomer at Anionic Lipid Bilayers: Implications for Membrane-Mediated Aggregation

**DOI:** 10.3390/ijms14036241

**Published:** 2013-03-19

**Authors:** Yan Jia, Zhenyu Qian, Yun Zhang, Guanghong Wei

**Affiliations:** State Key Laboratory of Surface Physics, Key Laboratory for Computational Physical Sciences (MOE), Department of Physics, Fudan University, 220 Handan Road, Shanghai 200433, China; E-Mails: 10210190018@fudan.edu.cn (Y.J.); 09110190008@fudan.edu.cn (Z.Q.); 0529009@fudan.edu.cn (Y.Z.)

**Keywords:** type 2 diabetes, human islet amyloid polypeptide, anionic palmitoyl oleolyohosphatidyl glycerol (POPG) bilayer, adsorption dynamics, binding orientation, peptide-lipid interaction, molecular dynamics simulations

## Abstract

Protein misfolding and aggregation cause serious degenerative diseases, such as Alzheimer’s and type II diabetes. Human islet amyloid polypeptide (hIAPP) is the major component of amyloid deposits found in the pancreas of type II diabetic patients. Increasing evidence suggests that β-cell death is related to the interaction of hIAPP with the cellular membrane, which accelerates peptide aggregation. In this study, as a first step towards understanding the membrane-mediated hIAPP aggregation, we investigate the atomic details of the initial step of hIAPP-membrane interaction, including the adsorption orientation and conformation of hIAPP monomer at an anionic POPG lipid bilayer by performing all-atom molecular dynamics simulations. We found that hIAPP monomer is quickly adsorbed to bilayer surface, and the adsorption is initiated from the *N*-terminal residues driven by strong electrostatic interactions of the positively-charged residues K1 and R11 with negatively-charged lipid headgroups. hIAPP binds parallel to the lipid bilayer surface as a stable helix through residues 7–22, consistent with previous experimental study. Remarkably, different simulations lead to the same binding orientation stabilized by electrostatic and H-bonding interactions, with residues R11, F15 and S19 oriented towards membrane and hydrophobic residues L12, A13, L16 and V17 exposed to solvent. Implications for membrane-mediated hIAPP aggregation are discussed.

## 1. Introduction

The deposition of amyloid fibrillar aggregates is associated with the pathogenesis of many human diseases, such as Alzheimer’s, Parkinson’s and type II diabetes. For each disease, a specific protein is involved in amyloid fibril formation via a process that causes cytotoxicity, ultimately leading to degeneration [1,2]. The fibrillation process includes a lengthy lag phase during which a critical nucleus is formed and a rapid growth phase leads to the production of amyloid fibrils [1–3]. In type 2 diabetes mellitus, the major component of amyloid deposits is human islet amyloid polypeptide (hIAPP; also known as amylin), a 37-residue peptide hormone that is synthesized in pancreatic islet β-cells and co-secreted with insulin [4]. Studies on both model membranes and the cellular membrane show that the cytotoxicity of hIAPP is associated with peptide aggregation on the membrane surface by the formation of toxic oligomers [5] or by the growth of amyloid fibrils [6,7]. Increasing evidence shows that early formed hIAPP oligomers may be the most cytotoxic species [7–10]. The molecular mechanism by which this occurs is unknown, but likely involves the interaction of hIAPP with biological membranes, with a subsequent loss of integrity [10–13].

The hIAPP found in amyloid plaques is *C*-terminally amidated and contains a disulfide bond between Cys2 and Cys7 [14]. Experimental studies reported that monomeric hIAPP is predominantly unstructured in aqueous solution, although part of the chain, approximately residues 8–19, can transiently adopt an α-helical structure [15–17]. In the presence of lipid membranes, low-resolution structural studies have shown that hIAPP initially binds to the membrane in a helical state [18–20]. In an EPR (electron spin resonance) spectroscopy study of membrane-bound hIAPP at neutral pH, an α-helix is identified through residues 9~22 and is shown to be parallel to the membrane surface [21]. Recent NMR studies reported high-resolution surface-bound-helical structures of hIAPP in sodium dodecyl sulfate (SDS) micelles at both acidic (helix spanning residues 5–28) and neutral (a kinked helix formed by residues 7–17 and 21–28) pH [22,23]. After bound to lipid membranes at a high peptide:lipid ratio, aggregation of the peptide causes a cooperative conformational change from the helical conformation to the β-sheet amyloid form [18–20,24]. Despite extensive experimental studies, a detailed understanding of the first step of hIAPP-membrane interaction remains elusive. In particular, little is known about the adsorption process of hIAPP monomer from aqueous solution to the membrane surface and the orientation of hIAPP monomer when initially adsorbed at the membrane surface.

Complementary to experimental studies, molecular dynamics (MD) simulations can provide detailed structural information of peptides in different environments. A number of computational studies have explored the monomeric and oligomeric/fibrillar structures of full-length hIAPP [25–28] and its fragment [29–31] in aqueous solution. Due to the importance of the membrane in the aggregation and toxicity of hIAPP, studies on hIAPP in the membrane environment have been emerging. These studies have examined the oligomerization of hIAPP(12–18) and hIAPP(21–27) fragments [32], the monomeric structures of hIAPP1-25 and its S20G variant [33] and the ion channel activity of full-length hIAPP [34]. Recently, we have investigated the interactions of membrane-bound hIAPP monomers and dimers with anionic palmitoyl oleolyohosphatidyl glycerol (POPG) bilayers starting from a state where the hIAPP peptide are placed perpendicular to the plane of the membrane with its *N*-terminal 14-residues pre-inserted inside the POPG bilayer [35]. We have found that membrane-bound hIAPP monomers have a preference to move towards the membrane surface, while dimers can insert deep into the POPG bilayer. In the present study, we will investigate the adsorption orientation and conformation of full-length hIAPP monomer at the bilayer surface by performing all-atom MD simulations starting from a configuration where the peptide is placed in aqueous solution oriented parallel to the membrane surface. This study would provide theoretical insight into the first step of hIAPP-membrane interaction at the atomic level and will be useful for understanding the molecular mechanism of hIAPP aggregation at the membrane surface.

## 2. Results and Discussion

### 2.1. Adsorption of hIAPP Monomer from Aqueous Solution to the POPG Bilayer Is Mostly Initiated from the Positively Charged *N*-terminal Residues

**Figure 1:**
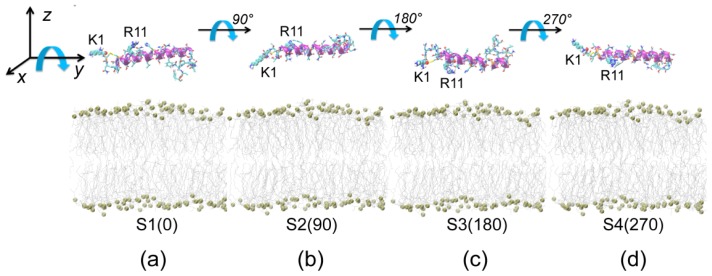
Four different initial states of simulated systems. In these four initial states, only the orientation of the peptide with respect to the bilayer is different. In Figure 1a, the side chains of residues K1 and R11 point away from the membrane surface. The orientations of human islet amyloid polypeptide (hIAPP) in (**b**), (**c**) and (**d**) are generated by rotating the hIAPP helix in (**a**) 90°, 180° and 270° around the axis of the helix (*y*-axis), respectively. The four different initial states are labeled as S1(0), S2(90), S3(180) and S4(270), according to the rotation angle. The minimum distances between the *N*-/*C*-terminus and the membrane surface are given in Table 1. The peptide is represented in the cartoon, with the helix (residues 5–28) in purple, the coil in orange and the other secondary structure in cyan. Amino acid residues K1 and R11 are in van der Waals (vdW) representation. The lipids are shown with hydrophobic tails in grey and phosphorus atoms as spheres. For clarity, counterions and water molecules are not shown.

**Figure 2:**
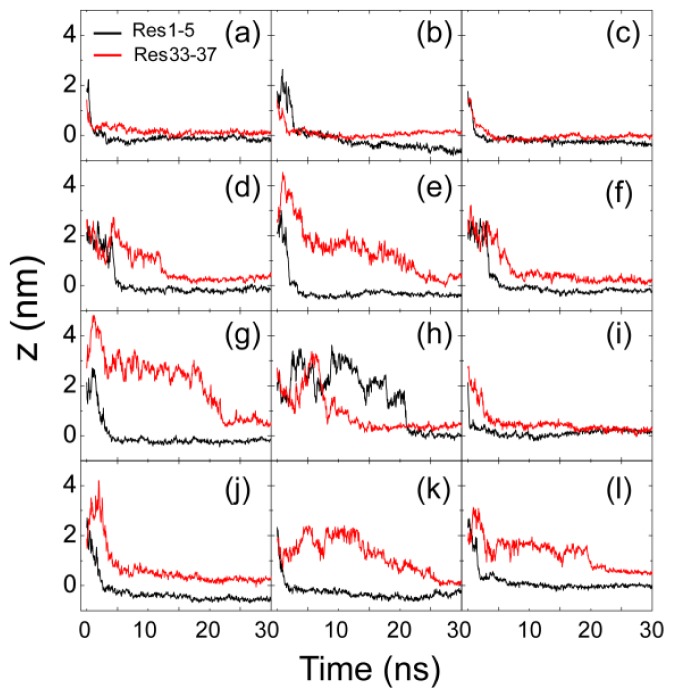
Time evolution of the minimum distance between the *N*-/*C*-terminus of hIAPP and the lipid bilayer for 12 independent molecular dynamics (MD) runs. The full simulation time is 120 ns for each MD run; for clarity, we show the first 30 ns only. The results in panels (**a**)–(**c**), (**d**)–(**f**), (**g**)–(**i**) and (**j**)–(**l**) are for the MD runs starting from S1(0), S2(90), S3(180) and S4(270), respectively. We use the minimum distance between the first/last five residues 1–5/33–37 (labeled as Res1–5/Res33–37) and the lipid bilayer to describe the distance between the *N*-/*C*-terminus and the bilayer surface.

**Figure 3:**
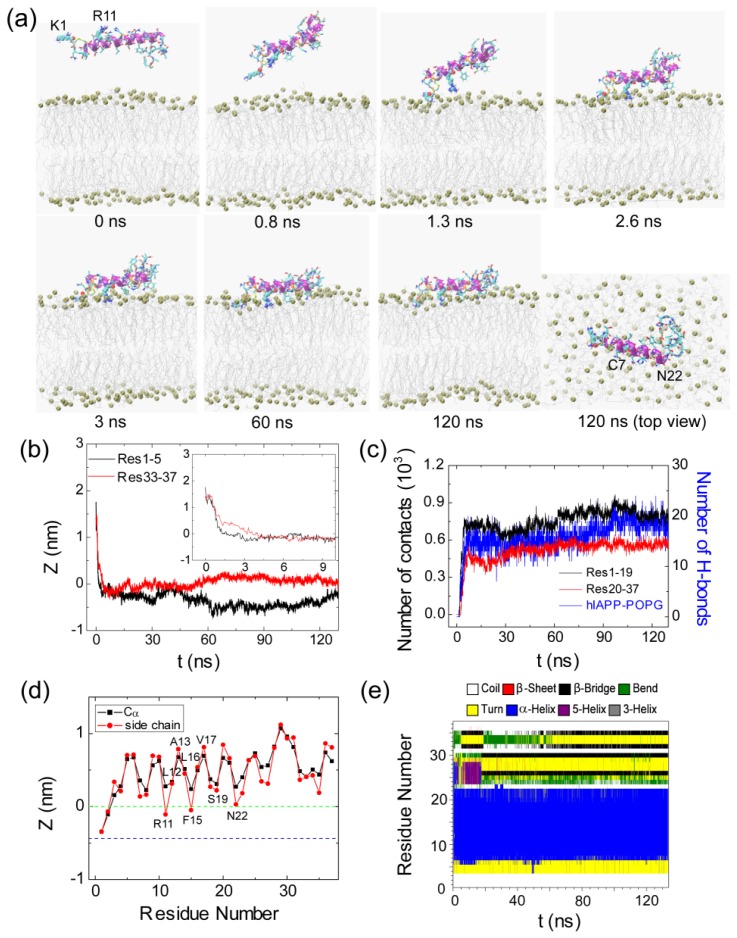
Detailed analysis of a representative MD trajectory starting from initial state S1(0). (**a**) Snapshots at seven different time points and the top view of the snapshot at *t* = 120 ns. Each snapshot is displayed using the same representations as those used in Figure 1; (**b**) Time evolution of the minimum distance between the *N*-/*C*-terminus of hIAPP and the lipid bilayer. In order to show clearly the initial adsorption process, we give in the inset the time evolution of the distance within the first 10 ns; (**c**) Time evolution of the number of contacts between *N*-/*C*-terminal residues 1–19/20–37 (Res1–19/Res20–37) and the palmitoyl oleolyohosphatidyl glycerol (POPG) bilayer and the number of hydrogen bonds formed between hIAPP and POPG lipids; (**d**) The time-averaged (last 20 ns) *z*-positions of the Cα atom (blue) and the side chain centroid (red) as a function of residue number. The dotted green and blue lines correspond to the headgroup phosphorus atom and the first carbon atom in the lipid tail of the sn-1 chain, respectively; (**e**) The secondary structure profile of hIAPP.

**Figure 4:**
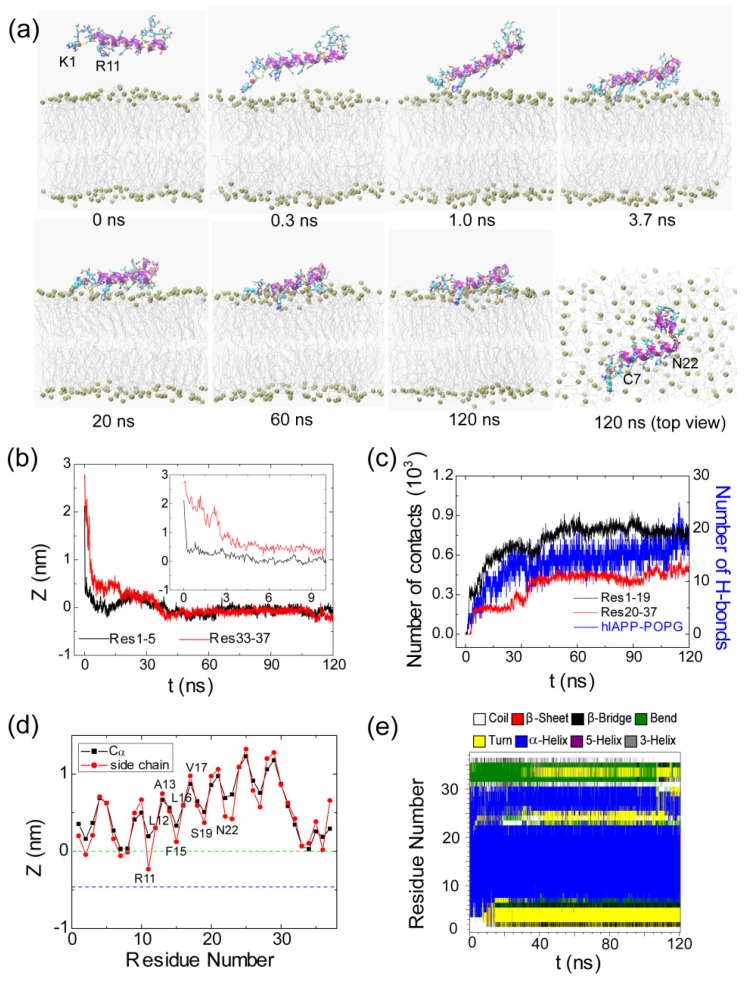
Detailed analysis of a representative MD trajectory starting from initial state S3(180). (**a**) Snapshots at seven different time points and the top view of the snapshot at *t* = 120 ns. Each snapshot is displayed using the same representations as those used in Figure 1; (**b**) Time evolution of the minimum distance between the *N*-/*C*-terminus of hIAPP and the lipid bilayer. In order to show clearly the initial adsorption process, we give the time evolution of the distance within the first 10 ns in the inset; (**c**) Time evolution of the number of contacts between *N*-/*C*-terminal residues 1–19/20–37 (Res1–19/Res20–37) and the POPG bilayer and the number of hydrogen bonds formed between hIAPP and POPG lipids; (**d**) The time-averaged (last 20 ns) *z*-positions of the Cα atom (blue) and the side chain centroid (red) as a function of residue number. The dotted green and blue lines correspond to the headgroup phosphorus atom and the first carbon atom in the lipid tail of the sn-1 chain, respectively; (**e**) The secondary structure profile of hIAPP.

We have performed 12 independent MD runs starting from four different initial states shown in Figure 1. The four initial states are labeled as S1(0), S2(90), S3(180) and S4(270). By monitoring the time evolution of the minimum distance between Res1–5/Res33–37 and the membrane surface (Figure 2), we observe that, in 10 out of 12 MD runs, the *N*-terminal residues, Res1–5, adsorb to the POPG membrane surface prior to *C*-terminal residues. We found that MD simulations starting from states with different initial orientations of the peptides with respect to the bilayer surface lead to quite similar adsorption behaviors. We give the results of two representative MD runs starting from S1(0) and S3(180) in Figures 3 and 4.

Figure 3 presents the snapshots at seven different time points and the time evolution of several parameters in the representative MD run starting from S1(0). These parameters include the Res1–5/Res33–37-bilayer distance, the number of atomic contacts between Res1–19/Res20–37 and the POPG bilayer, the number of H-bonds formed between hIAPP and the POPG bilayer and the secondary structure profile of each residue. To examine the binding orientation of the peptide, we also plot in Figure 3d the *z*-positions of Cα atom and the side chain centroid of each residue along the membrane normal. From the snapshots in Figure 3a, we see that the side chains of K1 and R11 point away from membrane surface in the initial state, where hIAPP is placed in water parallel to the POPG bilayer. Although the initial distance of Res1–5 (1.8 nm) to the bilayer surface is larger than that of Res33–37 (1.4 nm) (see the inset in Figure 3b), the side chain of the *N*-terminal residue K1 first moves toward the bilayer surface with respect to Res33–37 (see the snapshot at *t* = 0.8 ns). This is probably due to strong electrostatic attraction between the positively charged side chain of K1 and the negatively charged headgroups of POPG (see below for a more detailed discussion). Adsorption of K1 side chain to the headgroups of the POPG bilayer occurs at *t* = 1.3 ns. Binding of K1 to the POPG bilayer surface facilitates the other residues to be adsorbed to the membrane following an order from the *N*-terminal region to the *C*-terminal region. Adsorption of hIAPP peptide at the POPG bilayer takes place at *t* = 3 ns, with a minimum distance reaching 0.25 nm between Res1–5/Res33–37 and the POPG bilayer. Membrane adsorption is accompanied by the formation of H-bonds between hIAPP and the headgroups of POPG, as seen from Figure 3c. The following step is the rearrangement and the optimization of peptide side chains at the bilayer surface, which results in an increase in the number of atomic contacts (between Res1–19/Res20–37 and lipid bilayer) and the number of H-bonds between hIAPP and POPG. After *t* = 60 ns, we see a small fluctuation of the number of atomic contacts and the number of H-bonds. The peptide stays at the POPG bilayer surface parallel to the plane of the membrane, with the side chain centroids of K1, C2, R11 and F15 positioned below the phosphate group (see Figure 3d). The observation of these membrane-inserted *N*-terminal residues indicates that *N*-terminal residues 1–15 are bound to the membrane surface, consistent with an experimental study showing that the membrane binding site is largely localized to the *N*-terminal 1–19 residues of the peptide [36].

The results from the representative MD trajectory starting from the initial state of S3(180) are given in Figure 4. Similar analysis has been performed, as done for the MD run shown in Figure 3. Although the side chains of K1 and R11 point to the POPG bilayer at *t* = 0 ns (Figure 4a), the minimum distance between Res1–5/Res33–37 and the POPG bilayer is 2.1/2.7 nm (see the inset in Figure 4b), that is, there are no atomic contacts between hIAPP and POPG in the initial state. As seen from Figure 4b, the distance between Res1–5 and the bilayer decreases rapidly and reaches 0.5 nm at *t* = 0.3 ns, indicative of strong attraction between the Res1–5 and the POPG bilayer, while the distance between Res33–37 and the bilayer is still ~1.5 nm. The snapshot at *t* = 0.3 ns shows that it is the side chain of K1 that first approaches the membrane surface, revealing that the hIAPP-POPG interaction is initiated from the *N*-terminal positively charged residue, lysine. Interestingly, previous experimental studies suggested that *N*-terminal residues were involved in the initial interaction of hIAPP with lipids, in particular, negatively charged lipids [36–38]. Our MD simulation results provide atomic-level evidence for this hypothesis. The adsorption of K1 onto the membrane surface assists neighboring residues with interacting with the bilayer. We see that the *N*-terminal residue R11 is adsorbed to the membrane surface at *t* = 1.0 ns. At this time point, the Res1–5—POPG distance drops to 0.25 nm, while the Res33–37—POPG distance is still greater than 1.0 nm. This is followed by a continuous decrease of the Res33–37—POPG distance, reaching 0.25 nm at *t* = 3.7 ns (Figure 4b). This value of the Res33–37—bilayer distance indicates *C*-terminal residues that are adsorbed to the bilayer surface (see snapshot at *t* = 3.7 ns). After a local rearrangement of the residues at the surface, the whole peptide is adsorbed on the membrane surface (see the snapshot at *t* = 20 ns). As seen from Figure 4c, membrane adsorption is accompanied by an increase of the number of atomic contacts and the formation of H-bonds between hIAPP and POPG. With an increase of simulation time, the side chains of *N*-terminal residues start to insert into the bilayer. At *t* = 60 ns, the positively charged side chain of R11 is located slightly below the phosphate group (see the snapshot at *t* = 60 ns) and the peptide binds to membrane surface. In the left period of MD simulation, hIAPP remains in this position and the atomic contact/H-bond number remains almost constant. It is noted that the *N*-terminal residues bind more tightly to the bilayer than *C*-terminal residues, as reflected from the *z*-position of each residue along the membrane normal (see Figure 4d).

After characterizing the adsorption dynamics of hIAPP onto the POPG bilayer, we examine the conformation of hIAPP at the membrane surface. It is noted that the initial state of hIAPP used in our MD simulations is taken from a NMR-derived structure solved in SDS micelle at pH 4.6, while simulations are performed with a POPG bilayer at neutral pH. The snapshots in Figures 3a and 4a show that the structure of hIAPP adsorbed at the membrane surface consists of a stable helix from residues C7 to N22 and much less ordered *N*-terminal and *C*-terminal regions. Although the helical region in the initial state spans residues 5–28, the helix through residues 23–28 unfolds upon binding to the POPG bilayer, as seen from the time evolution of the secondary structure profiles shown in Figures 3e and 4e. The stable helix spanning residues 7–22 observed in our MD simulations is in good agreement with that reported in an EPR study of membrane-bound hIAPP at neutral pH [21].

### 2.2. hIAPP Monomer Has a Preferred Binding Orientation to the POPG Membrane Surface

**Figure 5:**
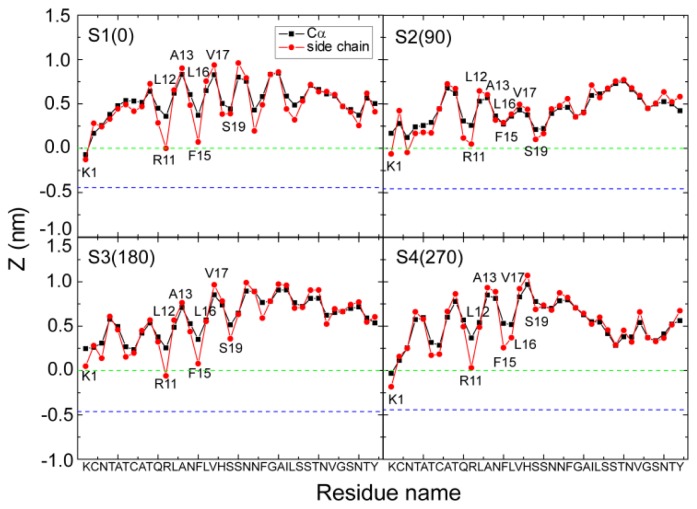
The *z*-positions of the Cα atom (blue) and the side chain centroid (red) of each residue. The results are for four different systems, S1(0), S2(90), S3(180) and S4(270). For each system, the *z*-position is an average of three different MD runs using the last 20 ns data. The dotted green and blue lines correspond respectively to the headgroup phosphorus atom and the first carbon atom in the lipid tail of the sn-1 chain. The region between the two lines is the headgroup region of the POPG bilayer. We observe that simulations with different starting orientations of the peptides relative to the bilayer surface lead to a similar binding orientation, with residues R11, F15 and S19 oriented toward the POPG bilayer surface.

It can be seen from Figures 3d and 4d that hIAPP binds to the headgroup region of POPG lipids, with most of the residues exposed to solvent. This result is consistent with a recent NMR study, which suggested that most of the residues of hIAPP were exposed to the solvent and the peptide was located close to the surface of the SDS micelle [23]. Figures 3d and 4d also show that the side chains of residues R11, F15, S19 and N22 in the helical region have smaller *z*-positions relative to their adjacent residues, indicating that hIAPP adopts a preferred orientation for interaction with the headgroups of the POPG bilayer. By detailed structural examination, we found that the side chains of R11, S19 and N22 form H-bonds with the headgroups of POPG lipids. To examine whether such a binding orientation is observed in all of the MD trajectories starting from the four different initial states, we have calculated the *z*-position of each residue (including the Cα atom and side chain centroid) in every MD run. Figure 5 presents the *z*-coordinate of each residue averaged over the three different MD runs for each initial state. Similar to the results given in Figures 3d and 4d, the side chains of residues R11, F15 and S19 in the helical region have a smaller *z*-position with respect to their neighboring residues. These results indicate that a similar binding orientation is achieved by hIAPP, with residues R11, F15 and S19 oriented towards the membrane surface, despite starting with different initial orientations of the peptides. The hydrophobic residues L12, A13, L16 and V17 are exposed to solvent. A previous EPR quenching experiment has shown a membrane-bound hIAPP monomer, with residues 9–22 forming an α-helix embedded in the bilayer 6–9 Å below the phospholipids headgroups. Residues Thr9, Leu12, Leu16 and Ser20 were suggested to face the hydrophobic core of the membrane, and the charged residues Arg11 and His18 are located at the level of the phospholipid headgroups [21]. The discrepancy in the membrane binding orientation of hIAPP monomer may be due to the different binding positions: the hIAPP helix lying on the bilayer surface in our MD simulation, while buried in the bilayer 6–9 Å below the phospholipids headgroups in the experiment [21]. Our simulations provide atomistic details of hIAPP conformation/orientation before insertion into the membrane, which will provide molecular insights into membrane-mediated hIAPP aggregation (see Subsection 2.5 for a detailed discussion). Except for the common binding orientation revealed from the MD runs starting from different initial states, we also found that the two positively charged residues, K1 and/or R11, have smaller *z*-positions than all the other residues, implying that these two residues play an important role on membrane binding of hIAPP.

### 2.3. Electrostatic Interaction Plays an Important Role on the Binding Behavior of hIAPP at Bilayer Surface

**Figure 6:**
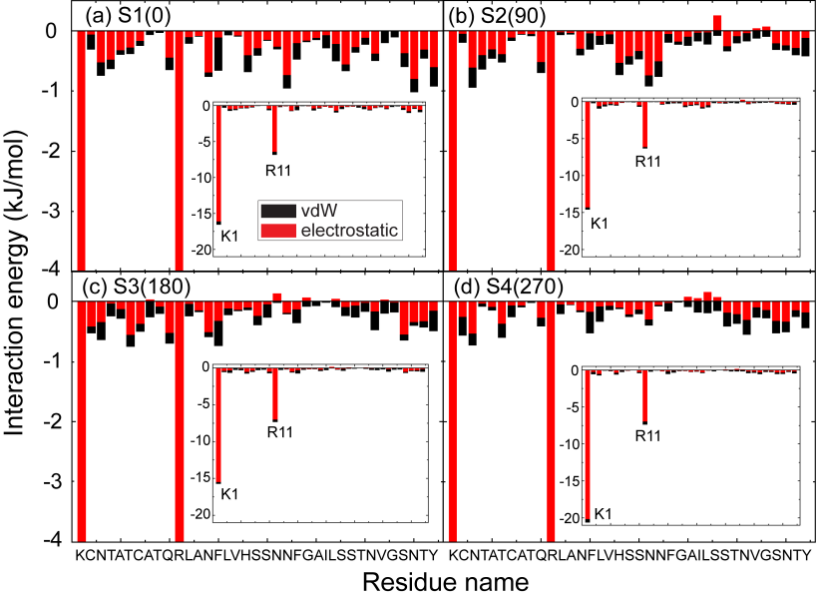
The interaction energy of each individual residue of hIAPP with POPG lipids (per lipid). The results are for four different systems, S1(0), S2(90), S3(180) and S4(270). For each system, the interaction energy is averaged over the last 20 ns of three MD runs. The residue-based interaction energy is decomposed into electrostatic and van der Waals (vdW) terms.

**Figure 7:**
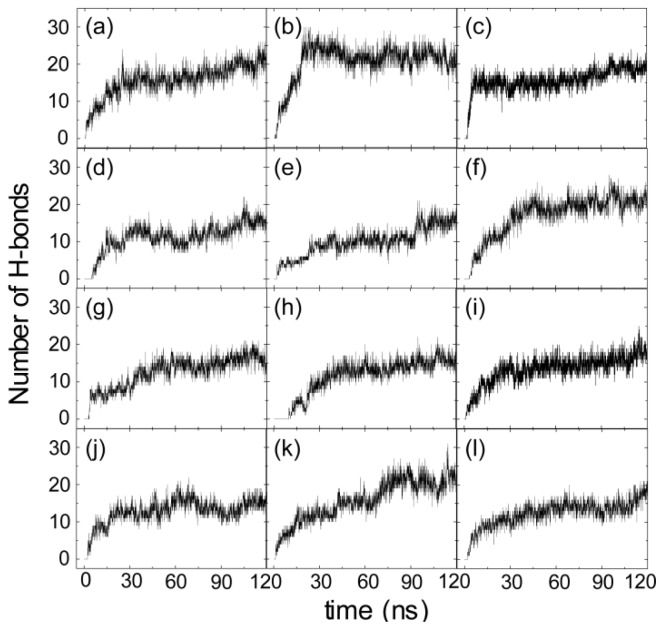
Time evolution of the number of hydrogen bonds formed between hIAPP peptide and POPG lipid for12 MD runs. The results in panels (**a**)–(**c**), (**d**)–(**f**), (**g**)–(**i**) and (**j**)–(**l**) are for the MD runs starting from S1(0), S2(90), S3(180) and S4(270), respectively.

To identify the important interactions for hIAPP binding at the POPG bilayer surface, we plot in Figure 6 the interaction energy between each amino acid residue and the POPG lipid bilayer (per lipid) for MD runs starting from the four different initial states. For each initial state, the interaction energy is averaged over the last 20 ns of three MD runs. In order to show the total contribution of K1/R11 to hIAPP-POPG interaction, we plot in the inset of Figure 6 the interaction energy using a different scale. The interaction energy is decomposed into electrostatic and van der Waals (vdW) components. We see that simulations with different starting orientations of the peptides with respect to the bilayer surface lead to the same results. First, electrostatic interaction is much stronger than vdW interaction, implying that the former plays a dominant role on the initial step of hIAPP-membrane interaction. Second, among all the residues, the positively charged residues, K1 and R11, interact most strongly with the anionic POPG bilayer. These two basic residues anchor the peptide to the bilayer by strong electrostatic interactions with charged lipid headgroups. The importance of electrostatic interactions between charged residues and lipid headgroups on the interaction of Alzheimer’s amyloid-β monomers/ protofibrils with zwitterionic POPC/POPE membrane surfaces has also been reported in recent MD studies [39,40]. Figure 6 also shows that the interaction energy of K1 with lipid is much larger than that of Arg11, as K1 carries 2+ out of the total 3+ charges of hIAPP. This result provides an explanation for the adsorption feature of hIAPP to the POPG membrane surface observed in our MD runs, that is, that the *N*-terminal residue K1 always first binds to the POPG bilayer (see Figures 3a and 4a) with respect to other residues. The interaction energy shown in Figure 6 demonstrates that the binding orientation of hIAPP at the POPG bilayer is mostly stabilized by electrostatic interactions between the *N*-terminal residues and the POPG headgroups. In addition, hydrogen bonds formed between peptides and lipids (see Figure 7) also play important roles.

### 2.4. hIAPP Monomer Adsorbed at Membrane Surface Displays Negligible Perturbation on Membrane Structure

**Figure 8:**
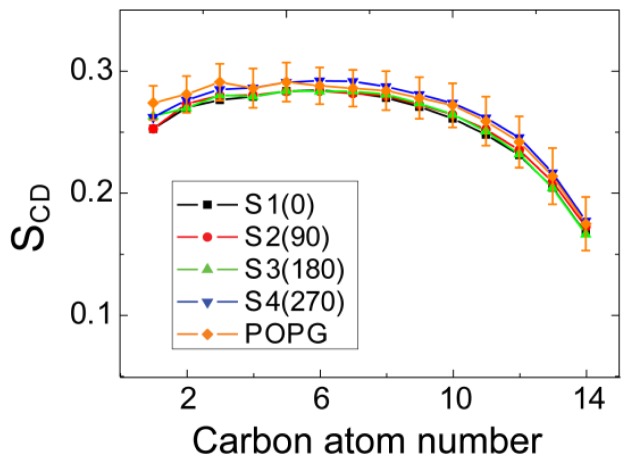
Order parameter, *S*_CD_, of lipid tails (sn-1 chain) for all of the systems. The order parameter is averaged over the last 20 ns of three MD runs for each system. For comparison, we also give the *S*_CD_ of the sn-1 chain obtained from the last 20 ns of a 100 ns MD run for pure POPG lipid bilayer.

It has been suggested that the toxicity of hIAPP and membrane disruption are associated with hIAPP-membrane interactions [10,24]. To examine whether binding of hIAPP monomer to the POPG membrane surface disrupts membrane structure, we first calculate the time-averaged thickness of POPG bilayer and the area per lipid over the last 20 ns of each MD run. The thickness of bilayer is estimated by the average of phosphorus-to-phosphorus distance. Our calculated value ranges from 4.30 to 4.45 nm for bilayer thickness and from 0.52 to 0.54 nm^2^ for area per lipid, in good agreement with the thickness of ~4.4 nm and area per lipid of ~0.54 nm^2^ for pure POPG bilayer obtained from 100 ns MD runs [35]. This indicates that binding of hIAPP monomer does not disrupt the integrity of the membrane structure. Then, we examine the effect of the hIAPP on the ordering of the lipid tail by calculating lipid tail order parameter, *S*_CD_. The value of *S*_CD_ is calculated using the formula *S*_CD_ = 0.5 〈3cos^2^θ − 1〉, where θ represents the angle of the C–H bond vector (in the simulation) or the C–D bond vector (in the experiment) with the bilayer normal. The angular brackets indicate an average over lipids and over time [41]. Figure 8 presents the average *S*_CD_ value of the acyl chain 1 (sn-1) of POPG lipids over three MD runs for each initial state. For comparison, *S*_CD_ of pure POPG lipid bilayer along with its error bar from a 100 ns MD simulation is also given in Figure 8. As seen from Figure 8, the *S*_CD_ values for MD runs from the four different initial states are within the error bar of the pure POPG lipid bilayer. The result of *S*_CD_, together with the calculated bilayer thickness and area per lipid, demonstrate that binding of hIAPP monomer at the POPG bilayer surface has negligible disturbance on the structure of the POPG bilayer. These results provide atomic-level evidence for the current viewpoint that membrane-bound hIAPP monomer does not cause membrane disruption [10,24,42].

### 2.5. Implications for Membrane-Mediated hIAPP Aggregation

Our MD simulations show that hIAPP binds to the POPG bilayer surface as a helical structure, with its hydrophobic residues exposed to solvent. These results have important implications for the membrane-mediated aggregation following the adsorption of hIAPP monomer at the POPG membrane surface. Previous experimental studies have reported that hIAPP aggregation and fibrillation are significantly accelerated in the presence of anionic lipid membrane [18,37,43]. Membrane-catalyzed hIAPP aggregation is believed to proceed first by the association of the helical regions of the peptide, followed by the formation of β-sheet structure in the disordered regions [24,44]. The orientation of hIAPP at the membrane surface is crucial to peptide-peptide interaction. Our MD simulations reveal an orientation of hIAPP at the POPG membrane surface, with the hydrophobic residues L12, A13, L16 and V17 exposed to solvent and residues R11, F15 and S19 oriented towards the membrane surface. It is apparent that the membrane-bound residues fall into the hydrophilic face of an amphipathic helix, whereas the solvent-exposed residues fall into the hydrophobic face. The membrane binding orientation indicates that the *N*-terminal region may play a crucial role in the self-association of hIAPP by controlling access to the putative dimerization interface on the hydrophobic face of the amphipathic helix. It is expected that the exposure of hydrophobic face to solvent would promote hIAPP aggregation by the enhanced orientation and the increased local concentration of hIAPP peptide. Based on our results, we propose a model for hIAPP aggregation at the membrane surface: hIAPP monomers first adsorb to the membrane surface as a helical structure; then, peptide self-association occurs through hydrophobic residues L12, A13, L16 and V17 and helix-helix association promotes conversion to the β-sheet structure.

## 3. Experimental Section

### 3.1. Peptide-Membrane System

POPG lipids are frequently used to model the anionic lipid bilayer [35,45,46]. Our model lipid bilayer consists of 2 × 64 anionic POPG lipids (*i.e.*, 64 lipids in each leaflet), and the initial coordinates are obtained from a previous computational study of a pure POPG lipid bilayer [47]. The amino acid sequence of hIAPP is KCNTATCATQ10RLANFLVHSS20NNFGA ILSST30NVGSNTY, with the Cys2 and Cys7 forming a disulfide bond. The side chains of Lys1 (Lys+) and Arg11 (Arg+) are charged in order to mimic the experimental neutral pH condition. The *N*-terminus is also charged (NH3+), while the *C*-terminus is amidated. hIAPP peptide carries three net positive charges at neutral pH.

It has been reported that hIAPP is predominantly α-helical when initially bound to the membrane [21–23]. As the time for folding a peptide in an explicit lipid membrane is on the order of a millisecond or second, it is still out of reach to sample the conformational transition from the random coil to helical structure at physiological temperatures. Therefore, we take one of the NMR-derived conformations (pdb ID: 2KB8) solved in SDS micelles at acidic pH as the initial structure of hIAPP [22], as done previously by us and others [33,35,48]. The core of the structure is an α-helix running from residues 5–28 with a distortion or kink near residues 18–22 [22]. hIAPP monomer is initially placed in aqueous solution parallel to the membrane surface. To allow the peptide to adjust its orientation before adsorption to the bilayer surface, the minimum distance between hIAPP and the bilayer surface is set to be ≥1.4 nm. We choose four different starting orientations of the peptides with respect to the bilayer, and the initial states are shown in Figure 1. In the first initial state, the side chains of residues K1 and R11 point away from the membrane surface (see Figure 1a). In the second (Figure 1b), third (Figure 1c) and fourth (Figure 1d) initial states, the orientations of the peptide are generated by rotating the hIAPP peptide in Figure 1a 90°, 180° and 270° around the axis of the helix (*i.e.*, *y*-axis), respectively. The four different initial states are labeled as S1(0), S2(90), S3(180) and S4(270), according to the rotation angle. The minimum distance between the *N*-/*C*-terminus and the membrane surface are given in Table 1. All of the hIAPP-membrane systems are fully solvated in a water box. Counterions (Na+) are added to neutralize the system.

### 3.2. MD Simulations

All of the MD simulations have been performed in the isothermal-isobaric (NPT) ensemble using the GROMACS 3.3.3 software package [49]. The POPG lipid is described with the Berger force field [50], and the peptide is described with the GROMOS-87 force field [51], as done in our previous work [35,46]. The water molecule is modeled by the simple point charge (SPC) model [52]. Bond lengths of peptides and lipids are constrained with LINCS [53], and water geometries are constrained with SETTLE [54], which allows an integration time step of 2 fs. Long-range electrostatic interaction is calculated using the particle mesh Ewald (PME) method [55] with a real space cutoff of 1.2 nm, as recommended for membrane simulations, especially for those involving charged lipids [56]. The van der Waals interaction is calculated using a cutoff of 1.4 nm. The temperature of the system is maintained at 310 K, above the gel-liquid crystal phase transition temperature 271 K of the POPG lipid membrane [57], as done recently by Tolokh *et al.*[58] Lipids, water, peptide and counterions are separately coupled to the temperature bath by weak coupling with a coupling constant of 0.1 ps [59]. The pressure is maintained at 1 bar by being weakly coupled to the pressure bath (with a coupling constant of 1.0 ps and a compressibility of 4.5 × 10^−5^ bar^−1^) [59] using a semi-isotropic scheme in which the lateral and perpendicular pressures are coupled separately. All MD simulations are performed using periodic boundary conditions in a rectangular box. Three independent 120 ns MD runs are carried out using different initial velocities for each initial state. A summary of the MD setup details is given in Table 1.

### 3.3. Analysis

We perform the MD trajectory analysis using our in-house-developed codes and the GROMACS facilities. The minimum distance between the *N*-/*C*-terminus of hIAPP and the bilayer surface is described using the distance between the first/last five residues 1–5/33–37 (labeled as Res1–5/ Res33–37) and the lipid bilayer. The interactions of the *N*-terminal residues 1–19 (Res1–19) and the *C*-terminal residues 20–37 (Res20–37) with the lipid membrane are estimated by the number of atomic contacts. The formation of hydrogen bonds (H-bonds) between hIAPP and the lipid bilayer is also monitored. Here, an atomic contact is defined when two non-hydrogen atoms come within 0.54 nm. A hydrogen bond is considered formed if the distance between N and O is less than 0.35 nm and the angle of N–H…O is greater than 150°. The position of each amino acid residue at the water-bilayer interface is described by the *z*-positions of the Cα atom and the side chain centroid of each residue averaged over the last 20 ns for each MD run. The secondary structure profile is given by the DSSP program [60]. The interaction energy of each amino acid residue with the POPG lipid bilayer (per lipid) is calculated, averaged over the last 20 ns of each MD run. In order to examine the effect of hIAPP on the ordering of the lipid tails, the order parameter, *S*_CD_, of the lipid acyl chain (sn-1) is calculated using the data generated in the last 20 ns of each MD simulation. The thickness of the POPG lipid bilayer is estimated by the average of the phosphorus-to-phosphorus distance [47]. All of the snapshots are displayed using the VMD program [61].

## 4. Conclusions

In this study, we have investigated the detailed binding process of hIAPP monomer at the anionic POPG bilayer surface by performing a series of all-atom MD simulations. A total of ~1.5-μs MD trajectories have been generated by starting from configurations in which the hIAPP monomer has four different initial orientations with respect to the POPG membrane surface. Although the hIAPP monomer is initially placed 1.4 nm away from and oriented parallel to the POPG bilayer surface, we find that the adsorption of hIAPP to the POPG bilayer is predominantly initiated from the *N*-terminal residues via electrostatic interactions, and the *N*-terminal positively-charged residue Lys1(+2) and Arg11(+1) make a dominant contribution to the adsorption process. This finding provides evidence for previous studies, which suggested that the *N*-terminal positive-charged residues were involved in the initial interaction of hIAPP with lipids [36–38]. Calculation of bilayer thickness, area per lipid and the lipid tail order parameter reveals that binding of hIAPP at the POPG bilayer surface has little effect on the integrity of the membrane and the lipid tail order. Although the initial structure of hIAPP is an NMR structure solved in detergent micelle at acidic pH, with residues 5–28 forming a helix, our MD simulations lead to a stable hIAPP helix spanning residues 7–22, in good agreement with previous EPR studies in anionic lipid bilayer at neutral pH. Moreover, hIAPP helix binds to the bilayer surface with the hydrophobic residues L12, A13, L16 and V17 exposed to solvent and residues R11, F15 and S19 oriented towards the membrane surface. This binding orientation is stabilized predominantly by electrostatic and H-bonding interactions between hIAPP and POPG headgroups. The solvent exposure of hydrophobic residues L12, A13, L16 and V17 would facilitate peptide-peptide association by hydrophobic interactions, which can in turn increase the rate of hIAPP aggregation and fibrillation, providing a possible explanation for membrane-accelerated hIAPP aggregation.

## Figures and Tables

**Table 1:** Set up details of MD simulations at 310 K. For each system, we describe the name of the system, the size of simulation box, the number of water molecules, the initial minimum distance between the *N*-/*C*-terminus and the lipid bilayer, the simulation time and the initial state of each MD run. We use the minimum distance between the first/last five residues 1–5/33–37 (labeled as Res1–5/Res33–37) and the lipid bilayer to describe the distance between the *N*-/*C*-terminus and the lipid bilayer. The superscript “a” in the sixth column means that the number in the bracket represents the number of the MD runs.

System	Simulation box size *a* × *b* × *c* (nm^3^)	# of water molecules	Initial distance Res1–5 (nm)	Initial distance Res33–37 (nm)	Simulation time (ns)^a^	Initial state
S1(0)	5 × 7 × 11	7,102	1.8	1.4	120 (3)	Figure 1a
S2(90)	5 × 7 × 12	8,325	2.1	2.6	120 (3)	Figure 1b
S3(180)	5 × 7 × 12	8,327	2.1	2.7	120 (3)	Figure 1c
S4(270)	5 × 7 × 12	8,311	2.3	1.9	120 (3)	Figure 1d
